# Eosinophilic dysplasia of the gallbladder: a hitherto undescribed variant identified in association with a "porcelain" gallbladder

**DOI:** 10.1186/1746-1596-1-15

**Published:** 2006-07-31

**Authors:** Oluwole Fadare, Steven D DeMartini

**Affiliations:** 1 Department of Pathology, Wilford Hall Medical Center, Lackland AFB, TX, USA; 2 Department of Pathology, University of Texas Health Science Center at San Antonio, San Antonio, TX, USA

## Abstract

Non-mass forming, neoplastic intraepithelial proliferations (dysplasia) represent the most well-accepted precursor lesions to gallbladder adenocarcinomas. They are typically small, localized, grossly unrecognizable lesions that have been identified in the epithelium adjacent to up to 79% of gallbladder adenocarcinomas. Morphologic variants that have been reported include flat, micropapillary, papillary and cribriform. We have recently encountered a morphologically distinctive, previously unreported lesion to which we have applied the designation *eosinophilic dysplasia*. This lesion was identified in a gallbladder with diffuse mural fibrosis and calcification (porcelain gallbladder). The dysplastic focus was confined to one tissue section, and was comprised of a localized true papilla [i.e with a fibrovascular core], measuring approximately 1.2 mm in greatest dimension and an adjacent, flat, 7-cell epithelial segment. These foci were lined by cells displaying significant nuclear enlargement [1.5–4 times the adjacent benign cells], nuclear pleomorphism, occasional multinucleation, hyperchromasia and nuclear membrane irregularities. Nucleoli were present but inconspicuous. These cells also showed voluminous eosinophilic to granular cytoplasm, such that the overall nuclear-to-cytoplasmic ratio was generally not increased. The cells displayed diffuse and marked nuclear immunoreactivity for p53, and approximately 70% of the cells showed nuclear positivity for Ki-67. The cells were also positive for cytokeratin 7 and were entirely negative for carcinoembryonic antigen (CEA) and chromogranin A. The cells of the adjacent normal epithelium were positive for cytokeratin 7 and CEA, negative for p53 and chromogranin A and showed a Ki-67 labeling index of <10%. Marked overexpression of the p53 protein as well as its high proliferative index are strong arguments in favor of the dysplastic nature of this lesion. However, further studies are required to elucidate its true clinical significance and to determine whether or not its association with a porcelain gallbladder, as noted herein, is entirely fortuitous. However, such studies can only be performed with an increased recognition by practitioners of this distinctive variant.

## Clinical history

A 76-year-old man was admitted with a suspicion of small bowel obstruction. His symptoms subsequently resolved without any operative intervention. However, a computed tomographic scan showed evidence of an enlarged "porcelain" gallbladder. A decision was made to electively perform a cholecystectomy, which was accomplished laparoscopically. His postoperative course was uneventful.

## Materials and methods

Tissue sections were fixed in 10% neutral buffered formalin, processed, embedded in paraffin, further processed and stained with hematoxylin and eosin. For immunohistochemistry, selected 4-micron thick, formalin-fixed, deparaffinized and rehydrated sections were stained with antibodies to ki-67 (clone MIB-1, dilution 1:100, heat-induced epitope retrieval [HIER], DakoCytomation, Carpinteria, CA], carcinoembyronic antigen (CEA) (polyclonal, 1:200, Proteolytic epitope retrieval), cytokeratin 7 (OV-TL 12/30, 1:100, HIER, DakoCytomation), p53 (D07, 1:50, HIER, DakoCytomation), and chromogranin A (DAK-A3, 1:100, HIER, DakoCytomation). Assays were performed on a DAKO autostainer (DakoCytomation) based on the avidin-biotin complex method.

## Pathologic findings

Macroscopic evaluation of the gallbladder showed it to measure 11.0 × 3.5 × 3.5 cm, and with a white-tan irregular serosal surface. The gallbladder wall was diffusely thickened, measuring up to 0.5 cm in thickness and displaying several areas of gross calcification. The mucosal surface of the gallbladder was tan-yellow. Although several nonspecific irregularities were noted on the mucosal surface, there was no distinct mass lesion. No choleliths were present. Seventy-four tissue sections, which represented approximately 80% of the entire gallbladder, were routinely processed for microscopic examination.

## Microscopic

**Figure 1:**
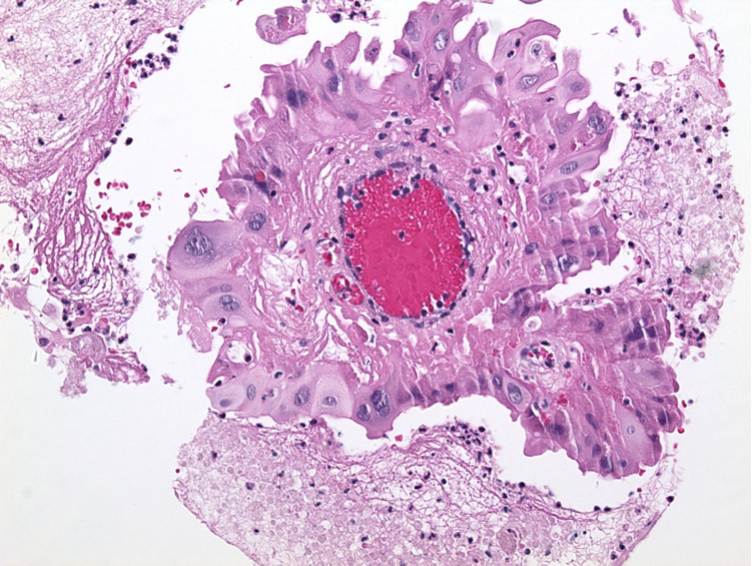
Focus of eosinophilic dysplasia, showing a papillae lined by cells with abundant eosinophilic cytoplasm (hematoxylin and eosin ×40).

**Figure 2:**
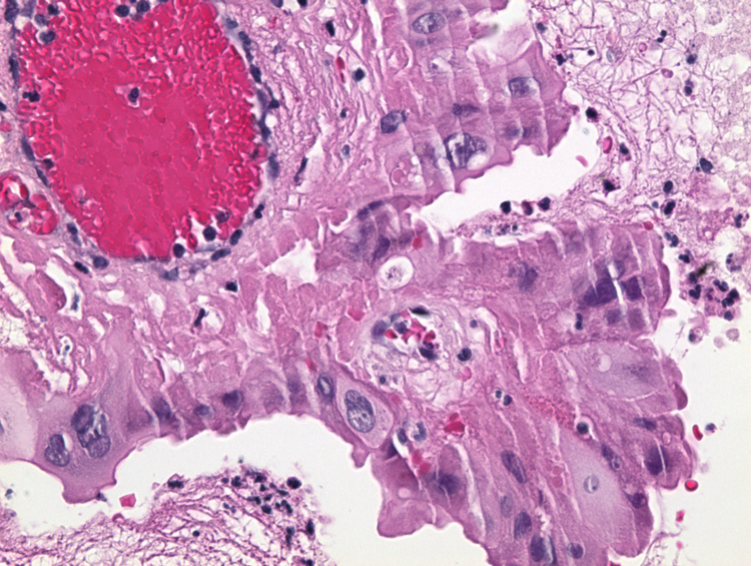
High power view of dysplastic focus showing nuclear pleomorphism. Note the multinucleation of cells in the lower left field (hematoxylin and eosin ×120).

**Figure 3:**
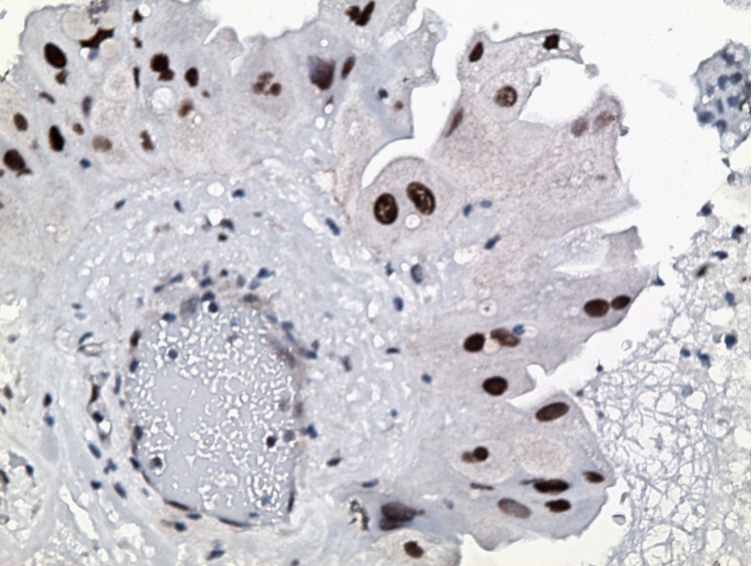
The dysplastic focus showed diffuse and marked immunoreactivity for p53 (immunoperoxidase ×160).

**Figure 4:**
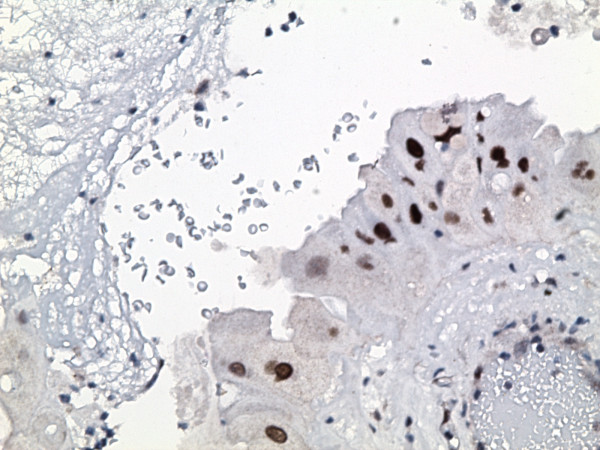
The dysplastic focus showed a high Ki-67 proliferative labeling index. (immunoperoxidase ×180)

The gallbladder showed diffuse mural fibrosis and dystrophic calcification (porcelain gallbladder). In approximately 50% of the sections examined, the mucosa was denuded. Where present, it was largely unremarkable. There were scattered foci of mucosal hyperplasia, as well as rare foci of intestinal and antral metaplasia. The dysplastic focus was confined to one tissue section, and was comprised of a localized true papilla [i.e with fibrovascular core], measuring approximately 1.2 mm in greatest dimension (figure 1) and an adjacent, flat, 7-cell epithelial segment. These foci were lined by cells displaying significant nuclear enlargement [1.5–4 times the adjacent benign cells], occasional multinucleation, nuclear pleomorphism, hyperchromasia and nuclear membrane irregularities (figure 2). Nucleoli were present but generally inconspicuous in most cells. These cells also showed voluminous eosinophilic to granular cytoplasm, such that the overall nuclear-to-cytoplasmic ratio was generally not increased. There was no nuclear stratification and mitotic figures could not be identified. Although cytoplasmic tinctural differences between cells occasionally resulted in an appearance of well-defined intercellular junctions, the latter were inconspicuous between most cells. The dysplastic cells displayed diffuse and marked nuclear immunoreactivity for p53 (figure 3), and approximately 70% of the cells showed nuclear positivity for Ki-67 (figure 4). The cells were also positive for cytokeratin 7 and were entirely negative for CEA and chromogranin A. The adjacent normal epithelium, including the hyperplastic foci were positive for cytokeratin 7 and CEA, negative for p53 and chromogranin A and showed a Ki-67 labeling index of <10%.

## Discussion

Epithelial dysplasia [ie a non-mass forming intraepithelial neoplastic lesion] is considered the most widely accepted precursor lesion to gallbladder carcinoma [1-3]. Several lines of evidence support this hypothesis. First, demographic studies indicate that patients with low and high-grade dysplasia are respectively 15 and 5 years younger than patients with invasive adenocarcinoma, suggesting a linear progression [4]. Second, dysplasia is more common in those portions of the gallbladder [body and fundus] where adenocarcinomas are most frequently localized (3). Third, the incidence of dysplasia is highest in those geographic areas with the highest incidence of invasive adenocarcinoma [5,6]. Fourth, dysplasia is found in the epithelium adjacent to a high percentage of adenocarcinomas [1-3]. Finally, some dysplasias share molecular alterations with invasive adenocarcinomas of the gallbladder [3].

The incidence of dysplastic lesions in cholecystectomy specimens is largely related to the geographic location of the patient population being studied, with figures ranging from 0.4%–5% in North American studies [7,8] to 13.5–13.6% in studies of the Chilean and Mexican populations [5,6]. They are more commonly identified in females, with a female to male ratio of 3:1 [9,10]. Dysplastic lesions are typically small, localized, grossly unrecognizable lesions. In one study, 68.6% of such lesions were <1 cm [9]. As noted previously, these lesions are frequently present in the epithelium adjacent to up 79% of invasive adenocarcinomas [6,11,12].

Dysplastic lesions are classified into low grade and high grade [carcinoma-in-situ] based on the presence of abnormally polarized nuclei and other prominent nuclear abnormalities in the latter. Reported morphologic variants include flat, micropapillary, papillary and cribriform variants [1,3]. In this report, we describe a morphologically distinctive and previously unreported variant that was characterized by abundant eosinophilic cytoplasm, nuclear pleomorphism and which was identified in association with a porcelain gallbladder.

The magnitude of the risk for malignancy conferred by diffuse gallbladder mural fibrosis and calcification (porcelain gallbladder) is controversial, with some studies showing an increased risk and others finding no such increase [13-15]. Therefore the gallbladder reported herein was extensively sampled [74 tissue sections], and the lesion described was identified only in one tissue section. However, other pathologic changes that have been suggested to play a role in the gallbladder carcinogenetic sequence, such as intestinal and gastric metaplasia, epithelial hyperplasia and cholecystitis, were present in many sections.

Given the aforementioned background of chronic inflammation, hyperplasia, fibrosis and calcification, the principal differential possibility that must be considered is that our lesion represents an unusual example of reactive atypia. Reactive atypia, in our experience, may show disorganized nuclei and increased eosinophilic cytoplasm. However, cells with reactive atypia will generally show prominent nucleoli, and only minimally enlarged nuclei. The exuberant eosinophilic cytoplasm noted herein will also be distinctly unusual for reactive atypia. The diffuse immunoreactivity for p53 in our lesion argues strongly in favor of its neoplastic nature. Wistuba et al [16] found overexpression of the p53 protein, as determined by immunohistochemistry, in 11 [32.4%] of 34 dysplasias. Neither normal epithelium nor metaplastic epithelium expressed p53 [16]. Similarly, Wee et al [17]. found that 28% of their dysplasias expressed p53. Normal epithelia in 38 gallbladders harboring various neoplastic, preneoplastic and nonneoplastic lesions were negative for p53, with the notable exception of one case associated with a gallbladder adenocarcinoma [17]. Kamel et al [18] found overexpression of p53 in 2 of 8 dysplasias. It is our opinion that the strong and diffuse overexpression of the p53 protein, in combination with morphologic features, supports our contention that this lesion is dysplastic. Additionally, the high proliferative index [70%] in the lesion, notably distinct from the background epithelium, further bolsters this contention. The minute nature of the focus precluded any further investigation (e.g electron microscopic) into the precise etiology of the cytoplasmic eosinophilia (e.g cytoplasmic accumulation of mitochrondria, lysosomes, ribosomes, protein, secretory vesicles etc).

In summary, we described herein a morphologically distinctive intraepithelial proliferation of the gallbladder which we have designated *eosinophilic dysplasia*. Further studies are required to elucidate the true clinical significance of this lesion and whether or not its association with a porcelain gallbladder, as noted herein, is entirely fortuitous. Meanwhile, practitioners should be aware of this distinctive variant when evaluating cholecystectomy specimens.

## Competing interests

The author(s) declare that they have no competing interests.

## Authors' contributions

SD and OF performed the pathologic evaluation of the specimen. OF wrote the initial version of the manuscript. Both authors have read and approved the final manuscript.
